# Top2 and Sgs1-Top3 Act Redundantly to Ensure rDNA Replication Termination

**DOI:** 10.1371/journal.pgen.1005697

**Published:** 2015-12-02

**Authors:** Kamilla Mundbjerg, Signe W. Jørgensen, Jacob Fredsøe, Ida Nielsen, Jakob Madsen Pedersen, Iben Bach Bentsen, Michael Lisby, Lotte Bjergbaek, Anni H Andersen

**Affiliations:** 1 Laboratory of Genome Research, Department of Molecular Biology and Genetics, Aarhus University, Aarhus C, Denmark; 2 Department of Cellular and Molecular Medicine, University of Copenhagen, Copenhagen N, Denmark; 3 Department of Molecular Medicine, Aarhus University Hospital, Aarhus N, Denmark; 4 Department of Biology, University of Copenhagen, Copenhagen N, Denmark; The University of North Carolina at Chapel Hill, UNITED STATES

## Abstract

Faithful DNA replication with correct termination is essential for genome stability and transmission of genetic information. Here we have investigated the potential roles of Topoisomerase II (Top2) and the RecQ helicase Sgs1 during late stages of replication. We find that cells lacking Top2 and Sgs1 (or Top3) display two different characteristics during late S/G2 phase, checkpoint activation and accumulation of asymmetric X-structures, which are both independent of homologous recombination. Our data demonstrate that checkpoint activation is caused by a DNA structure formed at the strongest rDNA replication fork barrier (*RFB*) during replication termination, and consistently, checkpoint activation is dependent on the *RFB* binding protein, Fob1. In contrast, asymmetric X-structures are formed independent of Fob1 at less strong rDNA replication fork barriers. However, both checkpoint activation and formation of asymmetric X-structures are sensitive to conditions, which facilitate fork merging and progression of replication forks through replication fork barriers. Our data are consistent with a redundant role of Top2 and Sgs1 together with Top3 (Sgs1-Top3) in replication fork merging at rDNA barriers. At *RFB* either Top2 or Sgs1-Top3 is essential to prevent formation of a checkpoint activating DNA structure during termination, but at less strong rDNA barriers absence of the enzymes merely delays replication fork merging, causing an accumulation of asymmetric termination structures, which are solved over time.

## Author Summary

Replication termination is the final step of the replication process, where the two replication forks converge and finally merge to form fully replicated sister chromatids. During this process topological strain in the form of DNA overwinding is generated between forks, and if not removed this strain will inhibit replication of the remaining DNA and thus faithful termination. In this study, we demonstrate that the cell has two redundant pathways to overcome topological problems during rDNA replication termination, one involving Top2 and the other involving the RecQ helicase Sgs1, in concert with Top3. In the absence of both pathways a checkpoint is activated in late S/G2 phase due to faulty replication termination at the strongest rDNA replication fork barrier (*RFB*). At less strong barriers termination is merely delayed under these conditions resulting in an accumulation of termination X-structures, which are solved over time.

## Introduction

During DNA replication termination two replication forks coming from opposite directions merge to form two fully replicated sister chromatids. The process is essential for correct transmission of the genetic information to the next generation, but it has so far attracted little attention in eukaryotes. In *E*. *coli*, replication terminates in a region defined by two sets of *Ter* sites bound by the polar terminator protein, Tus. This protein stops replication forks from one direction, but allows free passage of forks from the opposite direction. The Tus-*Ter* sites are organized so that they form a trap for replication forks, thereby ensuring termination in a region opposite *oriC* in the circular *E*. *coli* genome [1]. Polar replication fork barriers with a function in replication termination have also been identified in yeast. In *S*. *cerevisiae* the rDNA locus holds the Replication Fork Barrier sequence (*RFB*). This barrier binds the Fob1 protein, which mediates polar fork stalling at *RFB*, resulting in replication termination in this region [2]. In *S*. *pombe* polar replication fork barriers are found both at the rDNA and mating type loci, where replication fork arrest occurs at the termination sites *TER1-3* and *RFP4* [3] and at the Replication Termination Sequence 1 (*RTS*1) [4], respectively. At yeast barriers members belonging to the Pif1 family helicases, Rrm3 and Pif1 in *S*. *cerevisiae* and Pfh1 in *S*. *pombe*, have been demonstrated to play profound roles for fork stalling and fork merging [5,6,7].

71 termination regions (*TERs*) have been identified in the *S*. *cerevisiae* genome outside the rDNA locus in one of the first large-scale studies performed on this subject in eukaryotes [8]. A common theme to the sequences at the identified *TERs* was that they contained fork pausing elements and that the Rrm3 protein assisted fork progression through these zones. Furthermore, DNA topoisomerase II located to the *TERs* during the S and G2/M phases and prevented DNA breaks and genome rearrangements, suggesting that topoisomerase II plays a role to ensure proper replication termination.

Over the years several studies have implicated topoisomerase II in the final steps of replication. Early studies of the circular SV40 genome and the yeast-borne 2μ plasmid reported incomplete replication with nascent strands containing smaller or larger gaps upon inhibition of topoisomerase II activity [9,10,11,12]. Based on these studies a model was presented, suggesting that positive supercoiling accumulates between converging forks, leading to a rotation of the replisomes and formation of precatenanes behind the forks. As a consequence, genuine catenanes form following termination in the absence of topoisomerase II activity, since precatenanes are exclusively substrates for this enzyme [13,14].

The STR complex, which in *S*. *cerevisiae* consists of the Sgs1 RecQ helicase, topoisomerase III (Top3) and the Rmi1 protein, has mainly been studied in relation to its role downstream of homologous recombination (HR) [15,16,17]. Studies have provided evidence that the complex is involved in dissolution of double Holliday Junctions (dHJ) in a non-crossover process [18]. In this process Sgs1 is thought to disrupt local annealing between parental and nascent strands, thereby forming hemicatenanes, which can be decatenated by Top3 [16,18]. However, based on early results demonstrating an interaction between Sgs1 and topoisomerase II (Top2), as well as a chromosomal missegregation phenotype of *sgs1Δ* cells, components of the STR complex have also been proposed to play a role during late stages of replication [19]. In support of this, Marians and co-workers demonstrated that RecQ and topoisomerase III from *E*. *coli* in collaboration with the single-stranded DNA-binding protein SSB performed the resolution of a synthetic termination substrate *in vitro* [20]. The parental duplex DNA region between two stalled replication forks was here unwound by the RecQ helicase, while topoisomerase III simultaneously decatenated the resulting catenated strands, leading to gapped, but untangled termination regions. In line with this, Hickson and co-workers reported that the BLM-TOP3-RMI1-RMI2 complex localized specifically to ultrafine DNA structures, the so-called anaphase bridges, during M phase in human cells. It has been speculated that these bridges represent late replication intermediates that are resolved by the BLM-TOP3-RMI1-RMI2 complex [21,22,23]. Similarly, Sgs1 and Top3 were found to localize to anaphase bridges in budding yeast [24]. Together, these data indicate that RecQ helicases in concert with topoisomerase III play a role in late stages of replication besides their well-established role in the resolution of recombination structures.

Our aim with the present study has been to investigate if *S*. *cerevisiae* Top2 and Sgs1-Top3 act redundantly *in vivo* to ensure faithful replication termination and resolution of replicating chromatids. Our findings demonstrate that they do, and their redundant function in this process is restricted to the rDNA locus.

## Results

### Lack of Top2 and Sgs1-Top3 leads to robust checkpoint activation in late S/G2

**Fig 1:**
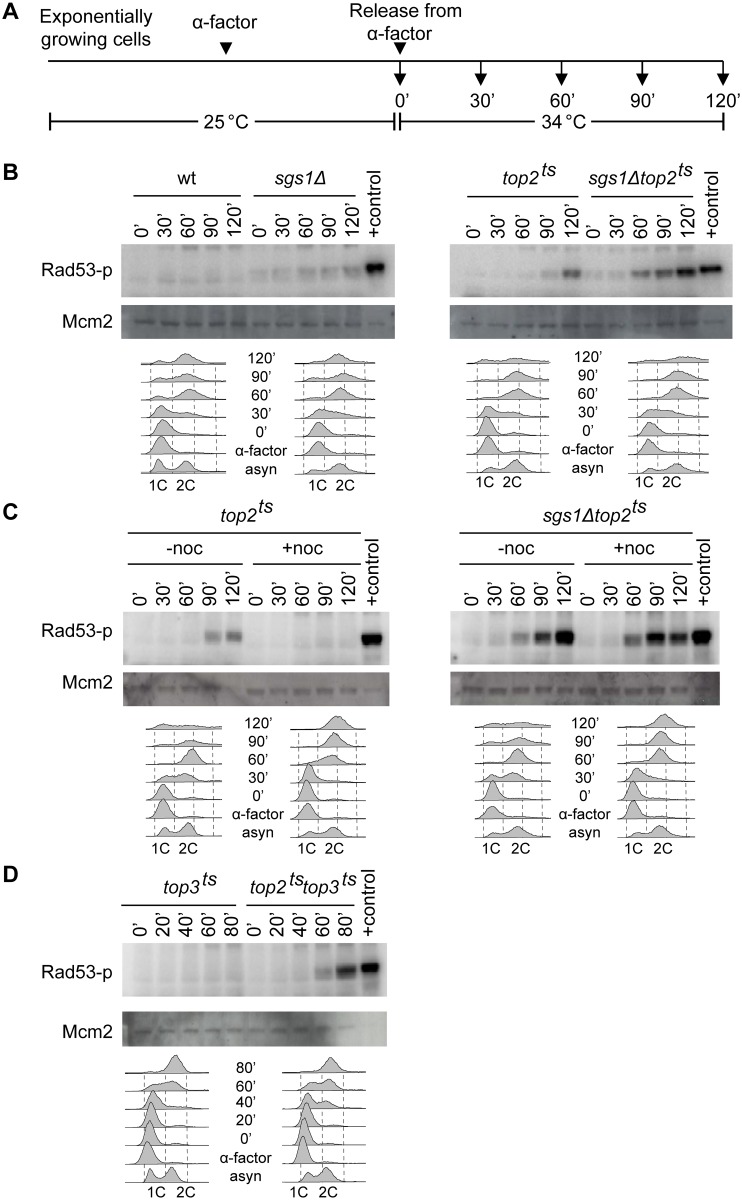
Loss of Top2 and Sgs1-Top3 leads to chromosome segregation-independent checkpoint activation. (A) Schematic illustration of the experimental setup. ISA analysis of Rad53 was performed with wt, *sgs1Δ*, *top2* ^*ts*^, and *sgs1Δtop2* ^*ts*^ cells (B), with *top2* ^*ts*^ and *sgs1Δtop2* ^*ts*^ cells treated with nocodazole (noc) as indicated (C), or with *top3* ^*ts*^ and *top2* ^*ts*^ *top3* ^*ts*^ cells (D). At the indicated time points after cells were released into YPD-media at 34°C, the restrictive temperature for *top2* ^*ts*^ (B and C) or at 37°C, a restrictive temperature for both *top2* ^*ts*^ and *top3* ^*ts*^ (D), samples were taken and processed for ISA analysis. The positive control (+control) represents ISA analysis on extract from MMS-treated wt cells. Mcm2 was used as a loading control. FACS profiles of samples taken throughout the experiment are shown below each strain. *1C* and *2C* indicate DNA content in G1 and G2, respectively, and *asyn*. refers to asynchronously growing cells.

Replicative stress and perturbations activate the S phase checkpoint pathway, which coordinates replication, repair, and cell cycling [25]. The Rad53 kinase is essential to this pathway and is phosphorylated, when the pathway is activated. If Top2 and the STR complex play redundant functions during final stages of replication, we expect that the absence of Top2 and Sgs1 will cause problems in late S/G2, which may activate Rad53. To test this hypothesis we monitored Rad53 phosphorylation in *sgs1Δtop2* ^*ts*^ cells using the *In Situ* Autophosphorylation (ISA) assay, which takes advantage of the autophosphorylation activity of Rad53, when it has been primed by upstream kinases [26]. As illustrated in the experimental setup presented in Fig 1A, yeast cells were grown at 25°C, synchronized in the G1 phase of the cell cycle with α-factor and released into the S phase, where samples were withdrawn at different time points and processed for checkpoint analyses. Release was at 34°C, the restrictive temperature for *top2* ^*ts*^. In the *top2* ^*ts*^ mutant, checkpoint activation was seen 120 minutes after release from α-factor (Fig 1B) in accordance with the previously identified function of Top2 in chromosome segregation [27,28]. Consistent with this, no checkpoint activation was seen in *top2* ^*ts*^ cells treated with nocodazole, which prevents the cells from entering mitosis by inhibiting microtubule polymerization (Fig 1C). Interestingly, the *sgs1Δtop2* ^*ts*^ double mutant showed robust checkpoint activation already 60 minutes after release into S phase (Fig 1B). To analyze whether this checkpoint was connected to failure in chromosomal segregation due to lack of Top2, *sgs1Δtop2* ^*ts*^ cells were treated with nocodazole. However, the robust checkpoint activation persisted in the presence of nocodazole and was thus independent of chromosome segregation (Fig 1C). As revealed by FACS analyses the observed checkpoint occurred in late S/G2 after bulk DNA synthesis had taken place.

Most known functions of Sgs1 are mediated through the STR-complex, where Sgs1 acts in concert with Top3 and Rmi1. To investigate if this was the case here, we tested a *top2* ^*ts*^ *top3* ^*ts*^ strain for checkpoint activation. The *top2* ^*ts*^ *top3* ^*ts*^ cells showed robust checkpoint activation 60 minutes after release into S phase (Fig 1D), illustrating that Sgs1 and Top3 are equally important in cells lacking Top2. Taken together, the data demonstrate that Top2 and components of the STR-complex function in late S/G2 to avoid the accumulation of checkpoint activating structures.

### Deletion of *FOB1* abolishes checkpoint activation in *sgs1Δtop2* ^*ts*^ cells

**Fig 2:**
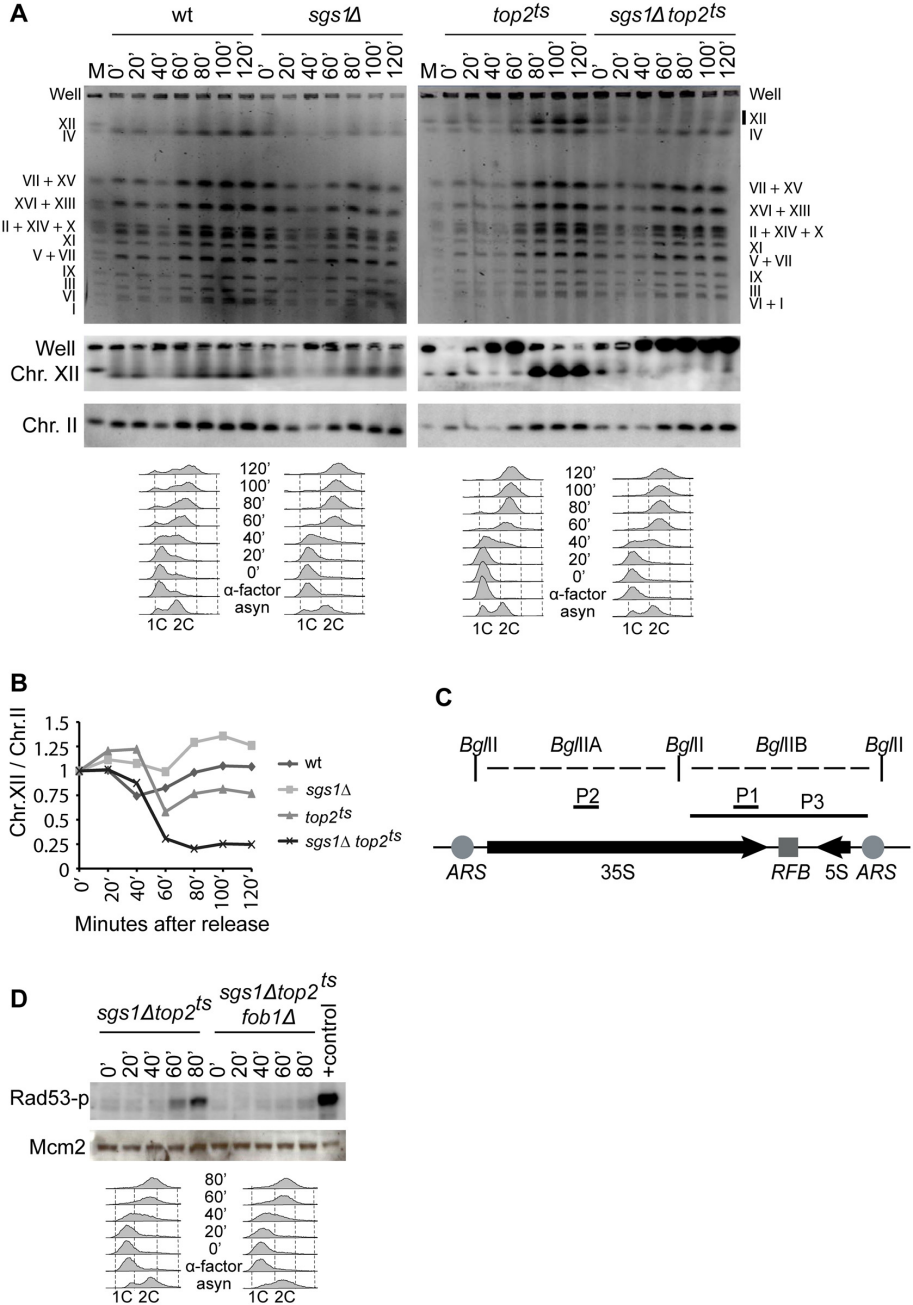
Checkpoint activation in *sgs1Δtop2* ^*ts*^ cells is dependent on Fob1. (A) The experimental setup was as shown in Fig 1A, except that cells were released at 34°C into nocodazole to block further cell cycling. Chromosomes were prepared from wt, *sgs1Δ*, *top2* ^*ts*^ and *sgs1Δtop2* ^*ts*^ cells and visualized after pulsed-field gel electrophoresis by EtBr staining (upper panels) and by Southern blotting with a probe (P1) specific for chr. XII (middle panels) or chr. II (lower panels). *M*. Chromosomal marker with indication of individual chromosomes to the left. Migration of individual chromosomes in the used strains is indicated to the right. FACS profiles of samples taken throughout the experiment are shown below each strain. (B) Quantification of the ratio of chr. XII to chr. II re-entering the gel. (C) Schematic illustration of the rDNA repeat unit in *S*. *cerevisiae*, indicating the 5S and 35S transcription units. *RFB*, Replication Fork Barrier. *ARS*, Autonomously Replicating Sequence. *Bgl*II restriction sites generating the *Bgl*IIA and B fragments as well as probes used for Southern blotting are indicated. (D) The experimental setup was as shown in Fig 1A. Proteins were isolated from *sgs1Δtop2* ^*ts*^ and *sgs1Δtop2* ^*ts*^ *fob1Δ* cells at the indicated time points and processed for ISA analysis of Rad53 as in Fig 1. FACS profiles are shown below each strain.

Pulsed-field gel electrophoresis (PFGE) has earlier been used to demonstrate replication abnormalities in yeast, because DNA structures associated with incompletely replicated chromosomes prevent gel entrance [29,30]. We therefore applied this technique to investigate if the checkpoint activating structures observed in *sgs1Δtop2* ^*ts*^ cells would affect the fate of the individual chromosomes during replication. EtBr stainings of the PFGs revealed a decrease in the intensity of the individual chromosomal bands 40 minutes after α-factor release (Fig 2A, upper panels), which correlated with active replication for all strains as revealed from the FACS profiles. However, after 60 minutes the intensity of the bands had increased, demonstrating completion of replication and that none of the strains experienced any overall defect in replication. However, in the *sgs1Δtop2* ^*ts*^ cells, chromosome XII (chr. XII), which holds the rDNA locus, never re-entered the gel after replication, as confirmed by Southern blotting with an rDNA specific probe (Fig 2A, middle panels). In contrast, chr. II, which was used as a control for the other chromosomes, re-entered the gel (Fig 2A, lower panels). A quantification of the amount of chr. XII relative to chr. II re-entering the gel is shown in Fig 2B. Thus, although bulk DNA synthesis seems to occur without major problems in *sgs1Δtop2* ^*ts*^ cells, either Sgs1 or Top2 is required to complete replication of chr. XII.

A major part of budding yeast chr. XII is made up of the rDNA locus, consisting of 100–200 repeats of a 9.1 kb unit holding the 35S and 5S rRNA genes. Each repeat holds a replication origin (*ARS*) and a replication fork barrier (*RFB*) sequence (Fig 2C), where the latter forms a unidirectional replication fork block, when bound by the Fob1 protein. This block specifically stalls replication forks coming from the direction of the 5S rRNA gene and inhibits head-on collision between replication and transcription of the 35S rRNA gene [31]. Stalling of the leftward moving fork at *RFB* also ensures that replication terminates within this region. The rDNA locus differentiates chr. XII from the remaining chromosomes and could thus be an obvious cause to the problems observed with this chromosome in *sgs1Δtop2* ^*ts*^ cells. To address if this was the case and if the underlying cause of the checkpoint activation observed in *sgs1Δtop2* ^*ts*^ cells was connected to Fob1-mediated unidirectional replication, we investigated the checkpoint response in a *sgs1Δtop2* ^*ts*^ *fob1Δ* triple mutant. Interestingly, the checkpoint signal was reduced to background levels in the triple mutant (Fig 2D). Thus, checkpoint activation in *sgs1Δtop2* ^*ts*^ cells is Fob1-dependent and therefore takes place as a result of events occurring at the rDNA locus.

It is well established that excessive amounts of ssDNA coated with the ssDNA binding protein RPA is a signal for checkpoint activation through the Mec1 kinase [32,33]. To investigate if the Fob1-dependent checkpoint activation observed in *sgs1Δtop2* ^*ts*^ cells was related to the formation of DNA structures containing ssDNA, foci analysis were performed with cells having the large subunit of RPA (Rfa1) tagged with CFP and Nop1 (a marker for the nucleolus [34]) tagged with RFP (S1 Fig). The results demonstrated that *sgs1Δtop2* ^*ts*^ cells experienced significantly more RPA foci and thus more ssDNA 60–100 minutes after α-factor release relative to wt cells and single mutants, and most of the foci had a perinucleolar localization (S1A Fig Furthermore, formation of the ssDNA was Fob1-dependent (S1B Fig). Thus, the Fob1-dependent checkpoint activation observed in *sgs1Δtop2* ^*ts*^ cells is associated with a Fob1-dependent formation of ssDNA at the rDNA locus. The data indicate that the checkpoint activating DNA-structures observed in *sgs1Δtop2* ^*ts*^ cells contain regions of ssDNA.

### Late S/G2 checkpoint activation correlates with appearance of X-structures at the rDNA locus

**Fig 3:**
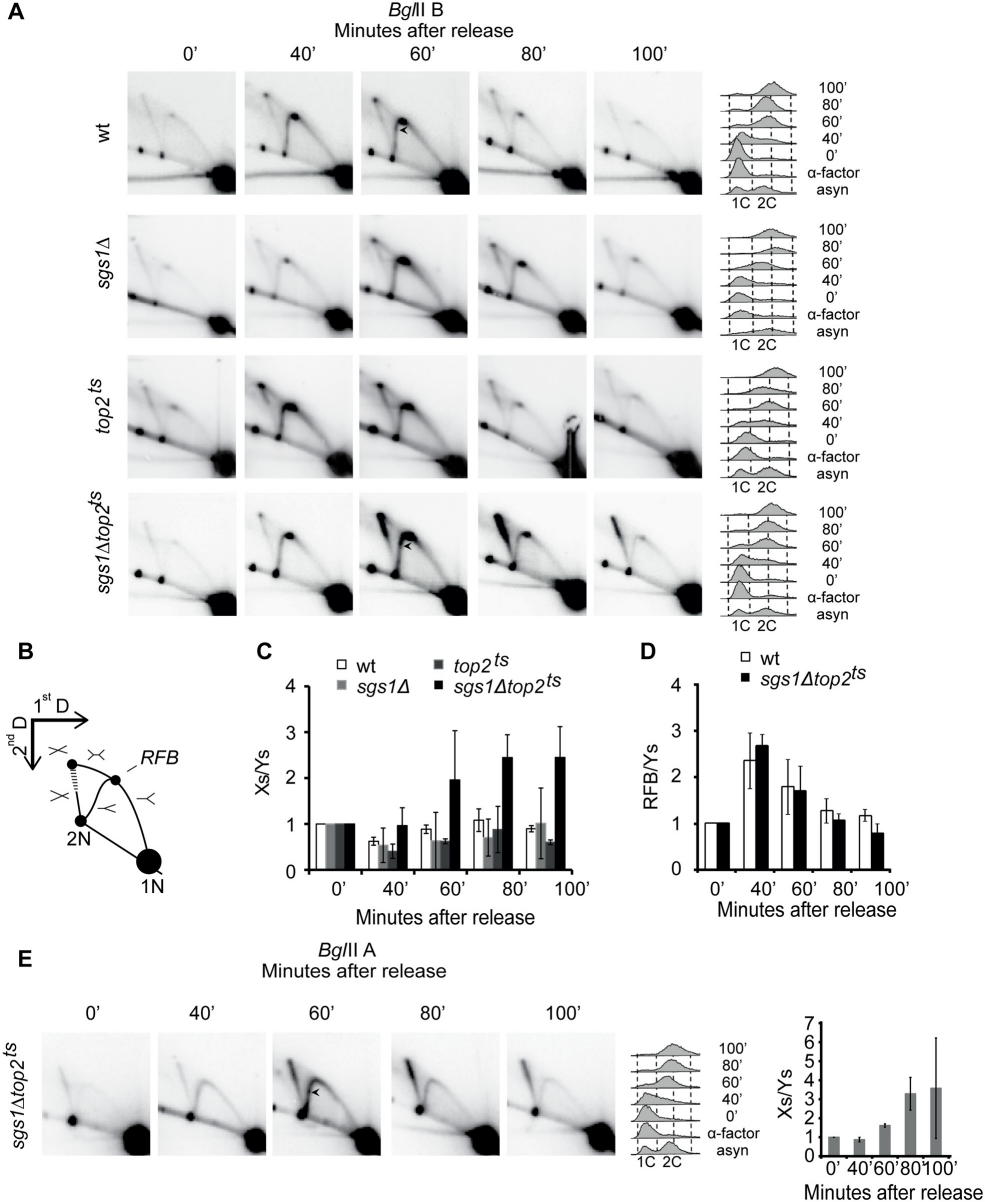
X-spike generating structures are formed in the rDNA in *sgs1Δtop2* ^*ts*^ cells. (A) The experimental setup was as in Fig 1A except that cells were released from α-factor at 34°C into nocodazole. Genomic DNA was isolated from wt, *sgs1Δ*, *top2* ^*ts*^ and *sgs1Δtop2* ^*ts*^ cells at the indicated time points after release, digested with *Bgl*II and subjected to 2D gel analysis and Southern blotting using the P1 probe recognizing the *Bgl*IIB fragment. FACS profiles of samples taken throughout the experiments are shown to the right. (B) Schematic illustration representing the expected migration behavior of replicating rDNA corresponding to the 4.577bp *Bgl*IIB-fragment encompassing the *RFB*. Forks converging at *RFB* are indicated by a horizontal symmetric “X” and makes up the “top of the X-spike”. The stippled area below represents asymmetric X-structures, which make up the “main X-spike” in *sgs1Δtop2* ^*ts*^ cells. (C) Quantification of Xs to Ys at the indicated time points, where the Xs/Ys obtained at time point 0 was set to 1. Error bars represent STDEV from two to four independent experiments. (D) Quantification of the *RFB* signal relative to all Ys at the indicated time points, where the ratio at time point 0 was set to 1. Error bars represent STDEV from three to four independent experiments. (E) Genomic DNA was isolated from *sgs1Δtop2* ^*ts*^ cells and treated as in (A) except that probe P2 recognizing the *Bgl*IIA fragment (Fig 2C) was used in the Southern blot. FACS profiles and quantifications of Xs to Ys are shown to the right. The arrowheads shown in (A) and (E) represent replication forks stalling at sites other than *RFB*.

To further analyze the nature of the checkpoint activating structures formed in the rDNA in *sgs1Δtop2* ^*ts*^ cells, we performed Neutral-Neutral two-dimensional (2D) gel electrophoresis with genomic DNA from *sgs1Δtop2* ^*ts*^ and control strains (Fig 3A). The *Bgl*II restriction sites used for generation of a DNA fragment with the *RFB* site centrally located (*Bgl*IIB) is shown in Fig 2C, and the migration of the replication structures obtained with this fragment in 2D gels is shown in Fig 3B. 40 minutes after release from α-factor, active rDNA replication occurred in all strains as demonstrated by formation of single and double Y-structures as well as structures generated due to replication fork blockage and convergence at *RFB* (Fig 3A). 80 minutes after α-factor release replication termination had occurred at most rDNA repeats in wt and single mutants as reflected by the disappearance of the majority of replication intermediates. However, a remarkable accumulation of DNA structures giving rise to a significant X-spike had taken place in *sgs1Δtop2* ^*ts*^ cells already 60 minutes after α-factor release, which coincided with the timing of checkpoint activation. Notably, the X-spike included the dot from symmetric X-structures representing forks converging at *RFB* as well as asymmetric X-structures extending the X-spike half way towards the 2N dot (indicated by the stippled area in Fig 3B). Quantification of the X-spike signal relative to the signal from Y-structures demonstrated an increase in the relative amounts of X-spike over time (Fig 3C), although the total amount of replication structures, including the X-structures, had decreased significantly after 100 minutes (Fig 3A). In contrast, *sgs1Δtop2* ^*ts*^ cells did not show an increase in *RFB* stalling relative to wt cells as revealed from a quantification of the *RFB* signal relative to the signal from all Y-structures (Fig 3D).

To investigate if X-structure formation was restricted to the area around *RFB* we analyzed the migration of replication structures obtained in the *Bgl*IIA fragment (see Fig 2C) covering most of the 35S transcription unit. Asymmetric X-structures were also formed in this fragment with the same timing and Xs/Ys ratio as in the *Bgl*IIB fragment (Fig 3E). In contrast, we did not see an accumulation of X-structures in *sgs1Δtop2* ^*ts*^ cells, when replication structures were analyzed in a fragment outside the rDNA, containing the *TER102* site on chr. I [8] (S2 Fig). Thus, lack of Sgs1 and Top2 causes an accumulation of X-structures in late S/G2, which seems to be restricted to the rDNA locus.

### Different structures account for checkpoint activation and X-spike formation

**Fig 4:**
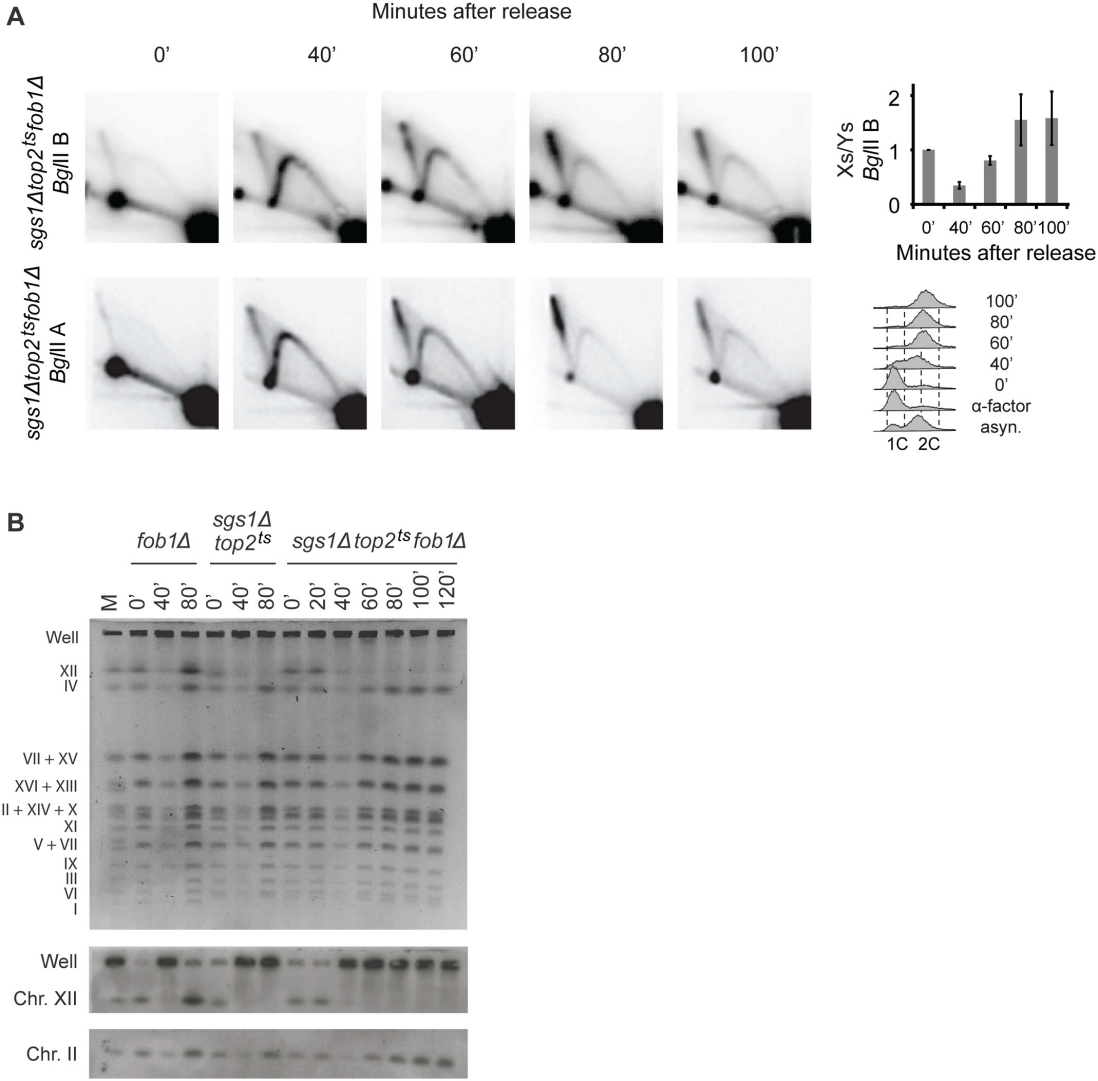
Asymmetric X-structures are formed independent of Fob1. (A) Genomic DNA was isolated from *sgs1Δtop2* ^*ts*^ *fob1Δ* cells at the indicated time points after release from α-factor and processed for 2D gel analysis and Southern blotting using either probe P1, recognizing *Bgl*IIB (upper panel), or probe P2, recognizing *Bgl*IIA (lower panel). Quantification of the Xs to Ys obtained with the *Bgl*IIB fragment is shown to the right, where the Xs/Ys at time point 0 is set to 1. Error bars represent STDEV from three independent experiments. FACS profiles are shown to the right. (B) Chromosomes were prepared from *fob1Δ*, *sgs1Δtop2* ^*ts*^ and *sgs1Δtop2* ^*ts*^ *fob1Δ* cells at the indicated time points after release from α-factor and visualized after pulsed-field gel electrophoresis by EtBr staining (upper panel) or by Southern blotting with a probe (P1) specific for chr. XII (middle panel) or chr. II (lower panel). *M*, Chromosomal marker with indication of individual chromosomes to the left.

Our data demonstrate that *sgs1Δtop2* ^*ts*^ cells show two strong characteristics, checkpoint activation and formation of X-structures, which are both connected to the rDNA locus. To investigate if the X-structures were the cause of checkpoint activation we took advantage of the Fob1-dependency of checkpoint activation and analyzed replication structures formed in the *sgs1Δtop2* ^*ts*^ *fob1Δ* triple mutant by 2D gel electrophoresis (Fig 4). Interestingly, the X-spike was still present in both *Bgl*II fragments. Thus, in contrast to the checkpoint activation (Fig 2D), all X-structures (except the symmetric X-structure formed due to forks converging at *RFB*) are formed independent of Fob1. Based on this we conclude that asymmetric X-structures are not responsible for checkpoint activation.

An investigation of chromosome migration in the *sgs1Δtop2* ^*ts*^ *fob1Δ* triple mutant by PFGE furthermore demonstrated that lack of Fob1 in the *sgs1Δtop2* ^*ts*^ strain was unable to suppress the migration defect of chr. XII (Fig 4), strongly indicating that the presence of X-structures in the rDNA is responsible for the inability of chr. XII to re-enter the gel after replication.

Taken together, our data are most consistent with a formation of two different DNA structures in *sgs1Δtop2* ^*ts*^ cells, a Fob1-dependent structure, which causes checkpoint activation, and a Fob1-independent structure causing the formation of the X-spike and the migration defect of chr. XII.

That checkpoint activation and X-spike formation are caused by different DNA structures was further supported by results obtained from experiments, where we investigated the sensitivity of the different structures to topoisomerase activity. In these experiments we reactivated Top2 in *sgs1Δtop2* ^*ts*^ cells (S3B and S3C Fig) and Top2 and Top3 in *top2* ^*ts*^ *top3* ^*ts*^ cells (S3D and S3E Fig) either before or after checkpoint activation and X-spike formation (25 or 60 minutes after release, respectively). We found that checkpoint activation and X-spike formation were inhibited upon early topoisomerase reactivation in both strains. In contrast, checkpoint activation was fully resistant to late reactivation in both strains, whereas X-spike formation was sensitive, showing a small reduction in the Xs/Ys ratio upon Top2 reactivation in *sgs1Δtop2* ^*ts*^ cells and a significant reduction upon reactivation of both topoisomerases in *top2* ^*ts*^ *top3* ^*ts*^ cells.

### Checkpoint activation and formation of X-spikes occur independently of homologous recombination

**Fig 5:**
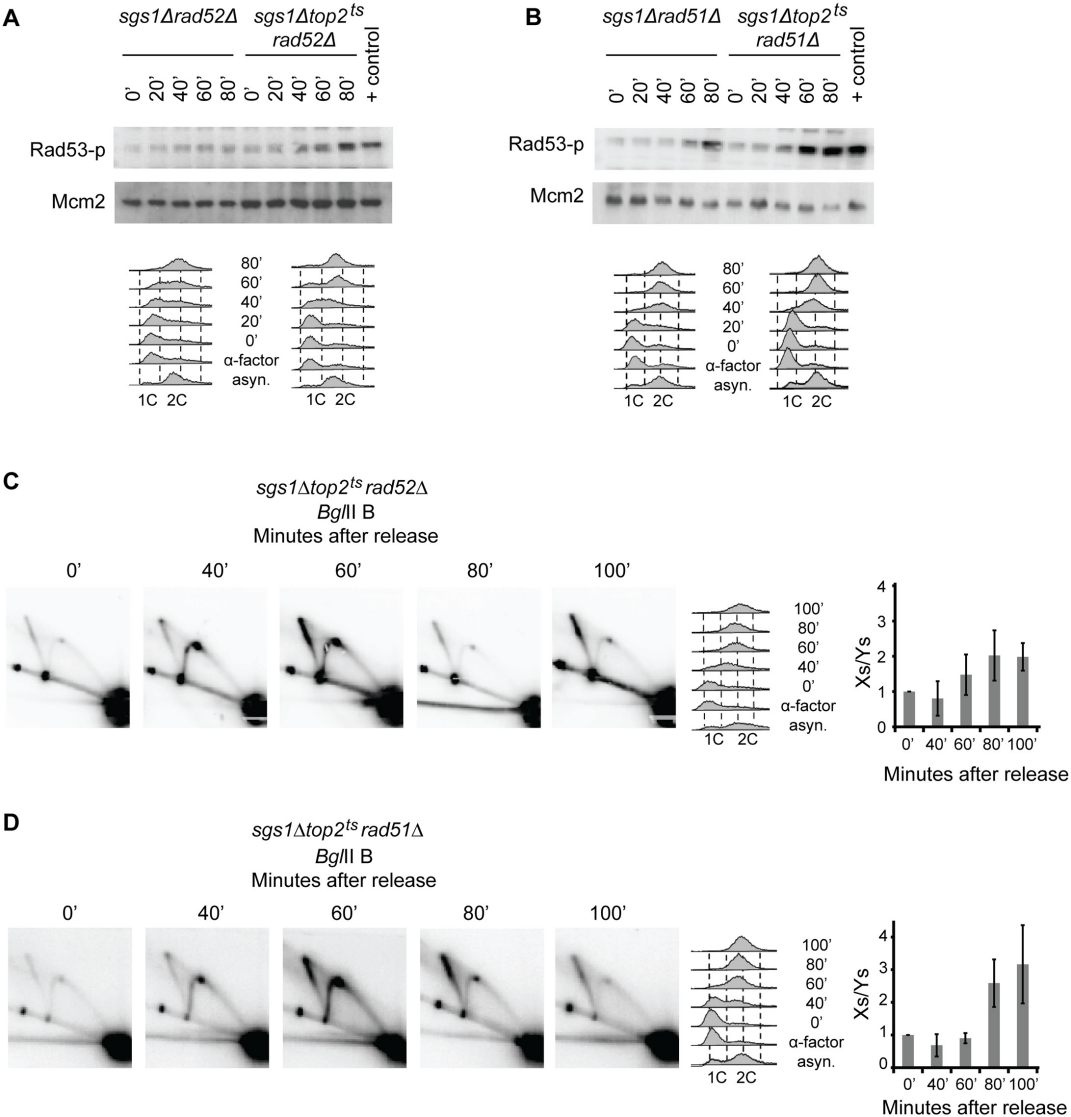
Structures causing checkpoint activation and X-spike formation in *sgs1Δtop2* ^*ts*^ cells are formed independently of homologous recombination. ISA analysis of Rad53 was performed as in Fig 1 with *sgs1Δrad52Δ* and *sgs1Δtop2* ^*ts*^ *rad52Δ* cells (A) or with *sgs1Δrad51Δ* and *sgs1Δtop2* ^*ts*^ *rad51Δ* cells (B). FACS profiles of samples taken throughout the experiments are shown below. Genomic DNA was isolated from *sgs1Δtop2* ^*ts*^ *rad52Δ* (C) or *sgs1Δtop2* ^*ts*^ *rad51Δ* cells (D) and processed for 2D gel analysis as in Fig 3 using the P1 probe recognizing the *Bgl*IIB fragment in the Southern blots. In the quantifications of Xs to Ys, the Xs/Ys obtained at time point 0 is set to 1. Error bars represent STDEV from three (in C) and two (in D) independent experiments.

The rDNA locus has previously been demonstrated to be highly recombinogenic with recombination hot spots located close to the *RFB* [35]. Furthermore, increased recombination activity has been observed within this locus both in *sgs1Δ* cells and *top2* ^*ts*^ mutants kept at semi-permissive conditions [29,36]. Since HR is visualized as X-structures in 2D gels [37] and since one of the important functions of Sgs1-Top3 is to dissolve dHJs downstream of Rad52-mediated HR [38], we wanted to investigate the relationship between HR and checkpoint activation as well as formation of X-structures in the *sgs1Δtop2* ^*ts*^ cells. We therefore deleted *RAD52* or *RAD51* in the *sgs1Δtop2* ^*ts*^ strain to investigate if lack of HR would suppress the checkpoint phenotype of *sgs1Δtop2* ^*ts*^ cells (Fig 5A and 5B). Although single and double mutants with *rad52Δ* (Fig 5A and S4 Fig) or *rad51Δ* (Fig 5B) showed a slight increase in basal checkpoint activation as expected, robust checkpoint activation was observed in the two triple mutants. This demonstrates that the checkpoint activation observed in *sgs1Δtop2* ^*ts*^ cells occurs independently of HR. Thus, checkpoint activation does not arise due to a HR structure left unresolved in the absence of the Sgs1-Top3 pathway.

X-spike generating DNA structures have been observed in several studies both at the rDNA locus [39,40,41,42] and in other chromosomal regions [8,42]. To investigate if the X-spike observed in *sgs1Δtop2* ^*ts*^ cells represents HR structures we investigated replication structures generated in the *sgs1Δtop2* ^*ts*^ *rad52Δ* (Fig 5C) and *sgs1Δtop2* ^*ts*^ *rad51Δ* (Fig 5D) triple mutants by 2D gel electrophoresis. In both mutants the X-spike was still present, and it persisted 100 minutes after α-factor release with a relative proportion of Xs to Ys similar to the one obtained in *sgs1Δtop2* ^*ts*^ cells. The same was true for the X-spike formed in the *Bgl*IIA fragment covering most of the 35S transcription unit (S5 Fig). Thus, like checkpoint activation, X-spike formation in *sgs1Δtop2* ^*ts*^ cells is not caused by unresolved recombination structures. This was further supported by the migration of the structures in 2D gels. Due to branch migration of junctions within HR structures these are expected to form X-spikes that extend with equal intensity over the entire spike, when experiments are performed in the absence of crosslinking agents, which is the case here [8]. In contrast, the X-structures in *sgs1Δtop2* ^*ts*^ cells only gave rise to signals in the upper half of the spike and often with a punctuate nature of the spike (see e.g. Figs 4 and 5C). By the same token it is unlikely that the X-structures in *sgs1Δtop2* ^*ts*^ cells represent hemicatenanes, which also form X-spikes in 2D gels [42].

### Checkpoint activation is related to replication termination at *RFB* whereas X-spike formation is related to termination at other rDNA barriers

**Fig 6:**
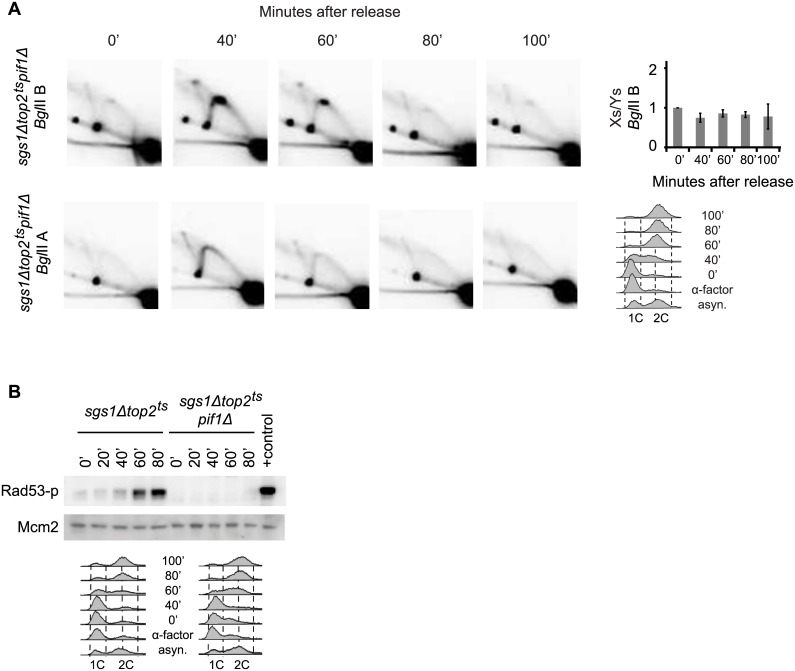
Stalling at *RFB* in the absence of Top2 and Sgs1 leads to checkpoint activation whereas stalling at other rDNA barriers causes X-spike formation. (A) Genomic DNA was isolated from *sgs1Δtop2* ^*ts*^ *pif1Δ* at the indicated time points after release from α-factor and processed for 2D gel analysis and Southern blotting using either probe P1, recognizing *Bgl*IIB (upper panel), or probe P2, recognizing *Bgl*IIA (lower panel). Quantification of the Xs to Ys obtained with the *Bgl*IIB fragment is shown to the right, where the Xs/Ys at time point 0 is set to 1. Error bars represent STDEV from three independent experiments. FACS profiles are shown to the right. (B) ISA analysis of Rad53 was performed as in Fig 1 with protein isolated from *sgs1Δtop2* ^*ts*^ and *sgs1Δtop2* ^*ts*^ *pif1Δ* cells.

Besides HR structures and hemicatenanes, forks converging during replication termination form X-structures. The X-spike in *sgs1Δtop2* ^*ts*^ cells could therefore represent termination structures and be indicative of a failure during replication termination. If this is the case converging forks at rDNA replication fork barriers other than *RFB* should be responsible for the asymmetric X-structures constituting the major part of the X-spike (indicated by the stippled area in Fig 3B) and faulty termination at *RFB* should cause checkpoint activation. Barriers other than *RFB* have been demonstrated in the rDNA repeat both at *ARS* and the 5S transcription unit in the *Bgl*IIB fragment as well as in the 35S transcription unit in the *Bgl*IIA fragment [6]. Dots representing forks stalled at some of these positions were visible both in wt and *sgs1Δtop2* ^*ts*^ cells (indicated by arrowheads in Fig 3A–3E). If asymmetric X-structures represent forks converging at these barriers and checkpoint activation is a result of faulty termination at *RFB* we speculated that a general weakening of all barriers would inhibit the accumulation of X-structures and diminish or abolish checkpoint activation. Rrm3 facilitates replication past replication fork barriers and has also been suggested to be involved directly in fork merging [8]. However, Pif1 has been demonstrated to counteract Rrm3 [6]. Therefore, if the DNA structures formed in *sgs1Δtop2* ^*ts*^ cells would represent termination structures formed at different rDNA barriers we would expect that a deletion of *PIF1* should reduce the formation of these structures either by directly facilitating fork merging at the barriers or by reducing fork stalling and thereby termination at these positions. To investigate this we deleted *PIF1* and analyzed replication structures generated in the *sgs1Δtop2* ^*ts*^ *pif1Δ* triple mutant by PFGE (S6A Fig) and 2D gel electrophoresis (Fig 6A and S6B Fig) to see the implications of a *PIF1* deletion for chr. XII migration and formation of asymmetric X-structures, respectively. Interestingly, chr. XII re-entered the PFG after replication in the *sgs1Δtop2* ^*ts*^ *pif1Δ* triple mutant and only wt levels of X-structures were observed in 2D gels for the triple mutant in both *Bgl*II fragments, consistent with X-structures representing termination structures. An alternative explanation for the Pif1 dependency of the asymmetric X-structures could be that *sgs1Δtop2* ^*ts*^ cells in the presence of Pif1 experience increased fork stalling at the different rDNA barriers, where the stalled forks are processed into X-structures in the mutant. However, we believe this is highly unlikely for several reasons. First, we do not observe increased stalling at *RFB* in *sgs1Δtop2* ^*ts*^ cells as expected if the cells in general show increased stalling (Fig 3D). Furthermore, processing of stalled forks into X-structures is expected to require HR, which is not involved (Fig 5). Finally, we observed an increased Xs/Ys ratio in *sgs1Δtop2* ^*ts*^ cells (Fig 3C), demonstrating an accumulation of X-structures rather than stalled forks, which indicates that processing of X-structures and not processing of Y-structures becomes the time limiting step in *sgs1Δtop2* ^*ts*^ cells. Based on this, it seems unlikely that forks stall more often in *sgs1Δtop2* ^*ts*^ cells than in wt cells. Rather the data suggest that when forks stalled at the different rDNA barriers are met by a fork coming from the opposite direction, fork merging becomes the time limiting step in *sgs1Δtop2* ^*ts*^ cells, thus resulting in an accumulation of termination X-structures.

To investigate, if a *PIF1* deletion affected checkpoint activation at *RFB* as expected if checkpoint activation is a result of faulty termination at this position, we investigated whether or not checkpoint activation occurred in *sgs1Δtop2* ^*ts*^ *pif1Δ* cells. As seen in Fig 6B checkpoint activation was fully abolished in the triple mutant. Thus, a deletion of either *PIF1* or *FOB1* inhibits checkpoint activation at *RFB*, whereas only a deletion of *PIF1* eliminates X-spike formation, consistent with a role of Pif1 at all rDNA replication fork barriers.

Taken together, the data suggest that lack of Top2 and Sgs1 becomes detrimental during replication termination at the rDNA locus in *sgs1Δtop2* ^*ts*^ cells. Thus, replication termination at *RFB* causes checkpoint activation in these cells, which can only be abolished if either Fob1 is fully removed or the barrier is “loosened” as expected in the absence of Pif1. In contrast, termination at less strong rDNA barriers is merely delayed in *sgs1Δtop2* ^*ts*^ cells and therefore accompanied by an accumulation of asymmetric termination X-structures, which decrease in amount over time and are sensitive to Top2/Top3 reactivation.

### Neutral-Alkaline 2D gels reveal accumulation of termination structures at *RFB* and other stalling sites at the rDNA locus in the absence of Sgs1 and Top2

**Fig 7:**
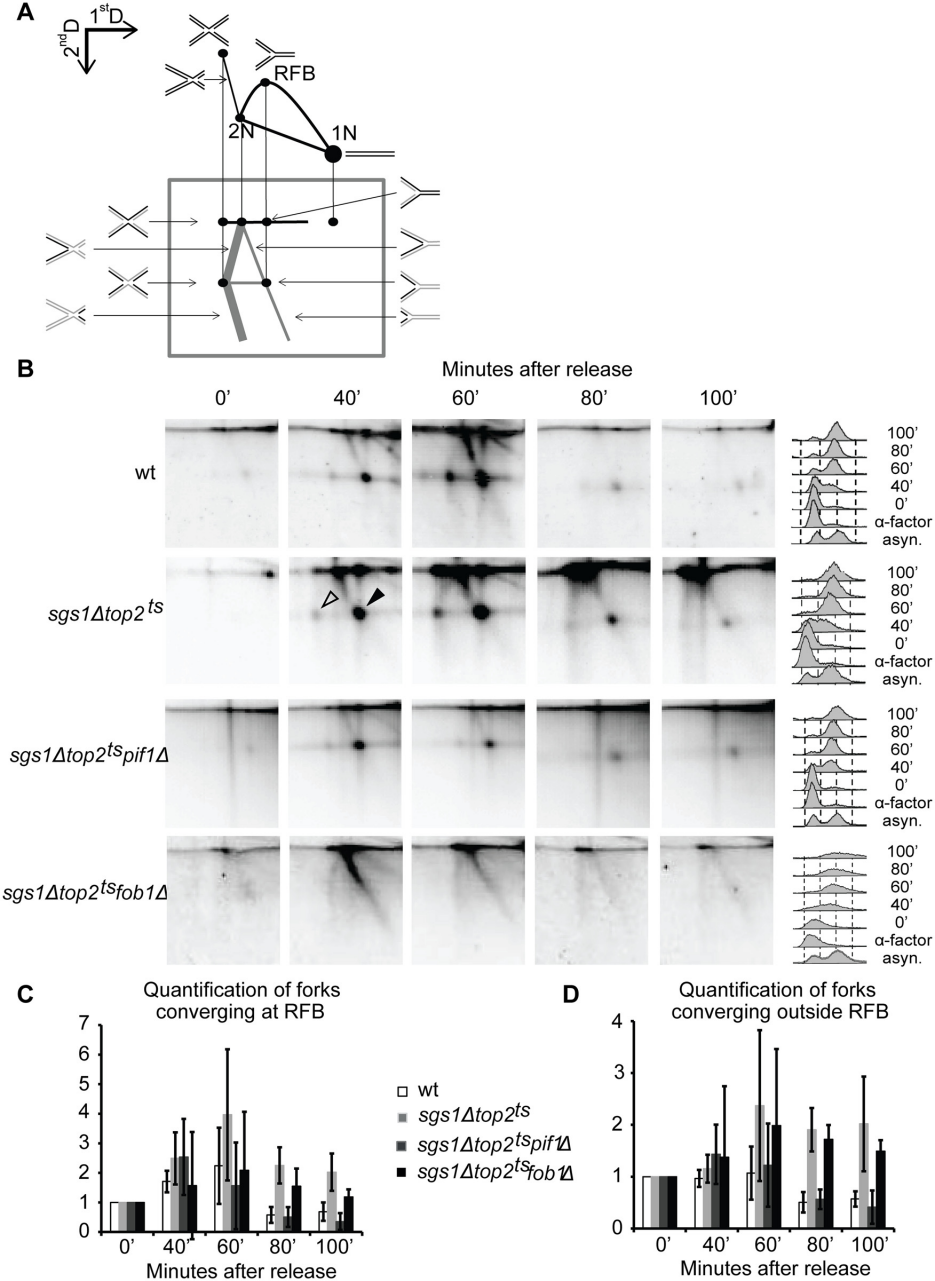
Replication termination structures accumulate at *RFB* and at other barriers in the rDNA in *sgs1Δtop2* ^*ts*^ cells. (A) Schematic illustration representing the expected Neutral-Alkaline 2D gel migration behavior of replicating rDNA corresponding to the 4.577bp *Bgl*IIB-fragment encompassing the *RFB* (grey box) with reference to the migration of the corresponding DNA in Neutral-Neutral 2D gels. Migration of the DNA strands shown with black lines is indicated. *RFB* as well as the positions of the 1N and 2N dots are indicated. (B) Genomic DNA was isolated from wt, *sgs1Δtop2* ^*ts*^, *sgs1Δtop2* ^*ts*^ *pif1Δ* and *sgs1Δtop2* ^*ts*^ *fob1Δ* cells at the indicated time points after release from α-factor, digested with *Bgl*II, and subjected to Neutral-Alkaline 2D gel analysis and Southern blotting using the P3 probe (Fig 2C). FACS profiles of samples taken throughout the experiments are shown to the right. (C) Quantification of forks converging at *RFB* at the indicated time points, where the signal at time point 0 is set to 1. Error bars represent STDEV from two to four independent experiments. (D) Quantification of forks converging outside *RFB* (“<”-smear) at the indicated time points, where the signal at time point 0 is set to 1. Error bars represent STDEV from two to four independent experiments.

Neutral-Alkaline (N-A) 2D gels have earlier been used to verify the presence of unsolved termination structures [7]. With this method, X-shaped molecules generated due to termination at *RFB* are separated from replication forks stalled at *RFB* in the first dimension due to differences in their molecular weight. After migration in the second dimension, where denaturation will separate DNA strands, both termination structures at *RFB* and forks stalled at *RFB* will consist of full length template strands as well as nascent strands of approximately half the size (Fig 7A). The template strands will thus form two dots located on the same horizontal line and the nascent strands will form two dots on a line below. Termination structures generated at positions other than *RFB* will form asymmetric X-structures. The template strands from these molecules will locate on the upper horizontal line, extending from the dot representing structures terminating at *RFB* towards the 2N dot. Besides full length parental strands, each asymmetric termination structure contains nascent strands of two sizes, which together make up the size of the parental strand. These strands will therefore in the second dimension form a “<” with legs emanating from the dot representing nascent strands from termination at *RFB* (Fig 7A, indicated by thick grey lines). Hemicatenanes and HR structures also form asymmetric Xs, but in contrast to termination structures both parental and nascent strands are full length and will locate on the upper horizontal line.

When DNA from *sgs1Δtop2* ^*ts*^ cells was analyzed with this method, spots representing nascent strands from replication forks blocked at *RFB* (black arrowhead) as well as nascent strands from X-shaped structures representing forks converging at *RFB* (open arrowhead) were revealed (Fig 7B, second row). The emergence of these spots 40 minutes after α-factor release correlated with the appearance of corresponding spots in wt cells (Fig 7B, first row) in agreement with the observations from Neutral-Neutral 2D gels (Fig 3). However, whereas the spots representing forks converging at *RFB* had disappeared to background levels in wt cells 80 minutes after release, they remained at a higher level in *sgs1Δtop2* ^*ts*^ cells (Fig 7C), although with a more diffuse appearance, strongly suggesting that termination at *RFB* was faulty. Furthermore, a smear extending as a “<” from the termination dot at *RFB* was present in wt and *sgs1Δtop2* ^*ts*^ cells 60 minutes after release, which remained in the mutant but disappeared in wt (Fig 7D). The presence of DNA fragments causing the “<”-smear shows that termination structures are formed at positions other than *RFB* in the rDNA and they remain for a prolonged time in *sgs1Δtop2* ^*ts*^ cells. In agreement with this, analysis of DNA isolated from the *sgs1Δtop2* ^*ts*^ *pif1Δ* and *sgs1Δtop2* ^*ts*^ *fob1Δ* triple mutants with this method demonstrated that the “<”-smear was absent in *sgs1Δtop2* ^*ts*^ *pif1Δ* cells as expected, but still present in *sgs1Δtop2* ^*ts*^ *fob1Δ* cells (except for the dots representing fork merging and stalling at *RFB*) (Fig 7B, third and fourth row and Fig 7D).

## Discussion

In this paper we demonstrate that cells lacking Top2 and Sgs1-Top3 show two strong characteristics, checkpoint activation and X-spike formation. Both are connected to the rDNA locus, appear during late stages of replication, and are independent of HR. Interestingly checkpoint activation is Fob1-dependent, whereas X-spike formation is not. In contrast, structures responsible for X-spike formation are sensitive to Top2/Top3 reactivation, whereas those responsible for checkpoint activation are not. Thus, two different structures are formed in *sgs1Δtop2* ^*ts*^ cells. This could either be due to a redundant function of Top2 and Sgs1-Top3 in two different processes or in a single process, having two different structural outcomes, when the enzymes are absent. Our results strongly suggest that it is the latter situation that is occurring, and that the process in which Top2 and Sgs1-Top3 are involved is replication termination. Thus, checkpoint activation occurs due to lack of Top2 and Sgs1-Top3 during replication termination at *RFB*, whereas X-spike formation is caused by a delay in replication termination at rDNA barriers other than *RFB*.

The results raise several questions. First, why do *sgs1Δtop2* ^*ts*^ cells have problems during replication termination and why do these cause checkpoint activation at *RFB* and only a delay in termination at other fork barriers? Furthermore, why do *sgs1Δtop2* ^*ts*^ cells show termination outside the normal *RFB* termination zone?

The finding that checkpoint activation occurs during termination at the strongest rDNA barrier whereas termination is merely delayed at other rDNA barriers when cells lack both Top2 and Sgs1-Top3, suggests that the problem experienced in the cells during termination is related to the nature of the barrier as well as to DNA topology. When a moving fork approaches a fork stalled at a barrier the topological tension between the forks increases and eventually leads to the formation of precatenanes behind the fork [13,14]. Several results have strongly suggested that the individual rDNA repeats are anchored to the nuclear membrane at *RFB* due to Fob1 interactions [43,44,45]. This fixation has been suggested to impose mobility constraints to the rDNA, and thus further increases the topological tension generated when forks converge at *RFB*. The structural consequence of the increased topological tension at *RFB* is speculative, but a checkpoint activating structure is finally formed, where either Top2 or Sgs1-Top3 can prevent formation of this structure as well as a deletion of either *FOB1* or *PIF1*. In this topologically tense region we propose that Top2’s role is to continuously decatenate precatenanes formed in the termination zone behind the replication forks. Top2-mediated decatenation will directly influence replication fork progression. However, the Fob1- and Pif1-dependency of checkpoint activation suggests that Top2 activity furthermore facilitates Rrm3-mediated Fob1 removal/fork merging as has been suggested earlier [8]. Our data demonstrate that Sgs1-Top3 work redundantly with Top2 in this process. In support of this, Rrm3 has been demonstrated to be synthetic lethal with Sgs1 and Top3 [46,47]. An obvious role of Sgs1 and Top3 would be to unwind and decatenate, respectively, the DNA between the two converging forks. In support of this the *E*. *coli* homologs of Sgs1 and Top3 have been demonstrated to perform this reaction *in vitro*, when acting on a DNA substrate holding two closely located forks, thus mimicking a late replication structure [20].

Cozzarelli’s lab has earlier demonstrated that the excess topological tension generated between replication forks promotes the formation of chickenfoot structures [48]. An equilibrium may thus exist between formation of precatenanes and chickenfoot structures. Another function of Sgs1-Top3 could therefore be to constantly revert chickenfoot structures to ensure replication fork progression and facilitate Rrm3-mediated Fob1 removal/fork merging together with Top2. In *sgs1Δtop2* ^*ts*^ cells the equilibrium may be shifted towards the formation of chickenfoot structures. We observed that ssDNA was generated at the rDNA locus in a Fob1-dependent manner with the same timing as checkpoint activation. If chickenfoot structures are the cause of checkpoint activation they could be subject to DNA end resection, generating a ssDNA overhang, which recruits RPA and mediates checkpoint activation through Mec1. We would not be able to discern these resected strands in N-A 2D gels due to the smear produced by replication termination at rDNA barriers other than *RFB*. The two suggested roles for Sgs1/Top3 are not mutually exclusive.

At the less strong barriers we expect that the topological tension generated when forks converge in the absence of Top2 and Sgs1-Top3 is allowed to slowly dissipate to more remote areas. This may be possible either because no anchorage is present to inhibit dissipation at these barriers or because the barriers are of a more transient nature. If the topological tension is lower than at *RFB*, chickenfoot structures may not be generated to an extent, where the amount of ssDNA exceeds the threshold required to trigger checkpoint activation. Rather, replication fork merging is merely delayed, causing an accumulation of asymmetric X-structures, which await dispersal of topological tension for final termination to take place. In correlation with this, the X-structures decreased in amount over time and were sensitive to late reactivation of Top2/Top3. Furthermore, X-structures were not visible in *pif1Δ* cells, indicating that the topological tension is easier to deal with when the barrier is more “loose”. This observation furthermore supports that the function of Top2 and Sgs1-Top3 also at the less strong rDNA barriers is to facilitate Rrm3-mediated barrier removal/fork merging.

If asymmetric X-structures in *sgs1Δtop2* ^*ts*^ cells represent termination structures, this means that termination to a great extent occurs outside the general *RFB* termination zone and thus that some replication forks pass *RFB* and are trapped at other barriers. Barriers other than *RFB* have been demonstrated in the rDNA including the 35S and 5S transcription units and the *ARS* element [6], where the nature of these barriers is unclear. Fork arrest was observed at some of these positions in wt as well as in the single and double mutants in the present study (Fig 3, arrowheads). Based on the migration of the asymmetric termination X-structures in 2D gels it seems as if mainly the transcription units act as fork barriers in *sgs1Δtop2* ^*ts*^ cells besides *RFB*. At these positions the barrier effect may be caused by collision of the fork with the transcription apparatus or with topological tension generated in excess in these regions due to lack of Top2 [49]. Besides the demonstration that barriers other than *RFB* exist in the rDNA, it has also been demonstrated that not all leftward moving forks are arrested at *RFB* despite the general unidirectional replication mode at the rDNA locus [50,51]. Thus, relative to wt cells *rrm3Δ* cells show increased fork arrest both at *RFB* and at the other rDNA barriers, demonstrating that passage through *RFB* to some extent occurs in wt cells. *RFB* escape has also been demonstrated in *pif1Δ* cells, where the fraction of DNA in leftward moving forks was increased 2.5-fold relative to wt cells [6]. Very interestingly, when these observations are taken into account, the increased termination we see in *sgs1Δtop2* ^*ts*^ cells at barriers other than *RFB* indicate that termination in general occurs outside *RFB* in wt cells, but only in *sgs1Δtop2* ^*ts*^ cells is termination at these positions delayed, resulting in the accumulation of termination X-structures. In support of this, the observation that the relative level of X-structures to Y-structures was very high and remained high in *sgs1Δtop2* ^*ts*^ cells suggests that termination rather than fork stalling becomes the rate limiting step in these cells. Furthermore, *sgs1Δtop2* ^*ts*^ cells showed no sign of increased fork stalling.

Our data strongly suggests that Sgs1-Top3 becomes essential for replication termination when Top2 is absent, but that this redundant action of Top2 and Sgs1-Top3 is restricted to the rDNA locus. In *sgs1Δtop2* ^*ts*^ *fob1Δ* cells, where the anchoring to the nuclear membrane of Fob1 bound rDNA repeats as well as unidirectional replication are abolished the situation is expected to mimic the situation outside the rDNA locus. However, under these conditions asymmetric termination X-structures were still observed in the rDNA, whereas we saw no accumulation of X-structures in *sgs1Δtop2* ^*ts*^ cells, when analyzing replication structures at *TER102* (S4 Fig) in correlation with earlier observations, where Top2, but not Top3, was found at termination sites outside the rDNA locus [8]. One explanation for this difference could be the high transcriptional activity at the rDNA locus. Transcription will increase the topological tension at the rDNA barriers which could create a need for Sgs1-Top3 besides Top2 and Rrm3 for efficient barrier dispersal/fork merging. Furthermore, it may well be that multiple copies are required to induce the robust cellular response observed in the rDNA. Thus, Top2 and Sgs1-Top3 may still play a role during termination outside the rDNA, but lack of Top2 and Sgs1-Top3 could cause less pronounced effects, which would be able to escape the detection limits of the assays employed in the present study.

## Materials and Methods

### Yeast strains

The employed strains were constructed using standard genetic techniques and are listed in S2 Table. All strains are derivatives of the original W303-1a.

### Media and growth conditions

Unless otherwise stated, cells were grown to logarithmic phase in YPD media. Synchronization in G1 was achieved by transferring cells to YPD (pH 5.0) containing α-factor (2 μg/ml, Lipal Biochem) followed by incubation at 25°C for 150 min. Additional α-factor (1 μg/ml) was added after 1 hour of incubation to avoid escape from G1. To release the cells from arrest, they were washed once in water and transferred to fresh, pre-warmed (34°C) YPD medium. G2 arrest was achieved by adding nocodazole (Calbiochem) to a final concentration of 15 μg/ml.

### Pulsed-field gel electrophoresis

Pulsed-field gel electrophoresis was performed as described in [52]. Cell cultures were grown to 3 x 10^7^ cells/ml, and approximately 1.8 x 10^7^ cells were cast into each plug to be run on the pulsed-field gel. The standard yeast genome size marker (Bio-Rad) was included on all gels. Gels were stained with ethidium bromide and transferred to Hybond XL membrane (GE Healthcare). Southern blotting was carried out using probes for chr. XII (Probe 1) and chr. II. Probes were amplified from purified yeast genomic DNA using 5’-CGCTTACCGAATTCTGCTTC and 5’-CTAGCATTCAAGGTCCCATT as forward and reverse primers, respectively, for chr. XII (Probe 1) and 5’-TCTCCGTCTTTAGTTGTTGC and 5’-GCCCTAGCAGTATTGCTTTG as forward and reverse primers, respectively, for chr. II. Experiments were performed 3–4 times with similar results.

### FACS analysis

Samples were taken for FACS analysis during the various experiments and processed as described in [53]. Samples were analyzed in a BD FACSCalibur.

### In situ kinase autophosphorylation (ISA)

All steps of the ISA were as described in [26], except that 5 μCi/ml [γ-^32^P] ATP was used. In short, protein extracts were generated from TCA-treated cells. For every sample, protein concentration was determined by Coomassie blue to allow loading of equal amounts of proteins on 10% SDS-polyacrylamide gels along with 5μl of a standard containing a known amount of MMS activated Rad53 (“+ control”). After gel electrophoresis proteins were transferred to PVDF filters (Immobilon-P, Millipore membranes). Filters were subjected to a denaturation/renaturation protocol before the autophosphorylation reaction was performed by incubating membranes in kinase buffer in the presence of [γ-^32^P] ATP. Dried filters were exposed on a Typhoon Trio+. After exposure, filters were re-probed with goat anti-Mcm2 (Santa Cruz) to check loading and allow comparison among different gels and mutants. Experiments were performed 2–3 times with similar results. MMS control (“+ control”): An Ay-120 culture (wt) with a density of 0.4 x 10^7^ cells/ml was treated with 0.1% MMS for ~60 minutes and harvested.

### Two-dimensional gel electrophoresis

Yeast genomic DNA was isolated from 1 x 10^9^ cells using Genomic-tip 20/G (QIAGEN) as described in [54]. After digestion with the *Bgl*II restriction enzyme (New England Biolabs) half of the purified DNA was subjected to Neutral-Neutral two-dimensional gel analysis as described in [55]. Southern blotting was carried out with the probes shown in Fig 2C, which were generated by PCR using genomic DNA as template. Probe 1 was generated as described above. For probe 2, recognizing the *Bgl*IIA fragment 5’-GTTTCTTTTCCTCCGCTT-3’ and 5’-ATCTCTTGGTTCTCGCAT-3’ were used as forward and reverse primers, respectively. For the probe near *TER102* on chr. I 5’-GAAGGTTCAACATCAATTGATTGATTCTGCCGCCATGATC-3’ and 5’- GCTTCCCTAGAACCTTCTTATGTTTTACATGCGCTGGGTA-3’ were used as forward and reverse primers, respectively.

For Neutral-Alkaline two-dimensional gel electrophoresis *Bgl*II digested DNA was run on a Neutral gel in the first dimension. In the second dimension the excised DNA was run on a 1.5% agarose gel in 50 mM NaOH plus 1 mM EDTA at 4°C [7]. Probe 3 (Fig 2C) used for southern blotting was made by PCR with 5’-CAGCCATAAGACCCCATC-3’ and 5’-GCAGTTGGACGTGGGTTA-3’ as forward and reverse primers, respectively, and genomic DNA as template.

### Quantification of DNA structures

The intensity of DNA structures was measured using QuantityOne software. For Neutral-Neutral 2D gels the relationship between either X-structures and Y-structures or the *RFB* dot and Y-structures was calculated for each time point. Unless otherwise stated, the ratio at the different time points was related to the ratio at the 0 minute time point to allow comparison between strains. For the Neutral-Alkaline 2D gels the signal of the *RFB* dot or the “<”-smear at the different time points was related to the signal at the 0 minute time point. For the PFG’s the ratio between chr. XII and chr. II re-entering the gel was calculated for each time point.

### Fluorescent foci analysis

All strains harbored the pWJ1321 plasmid encoding Nop1-RFP and were therefore grown in synthetic complete media without histidine [56]. Cells were synchronized in G1 by treatment with α-factor for 150 minutes and released into SC-his medium supplemented with 100 μg/ml adenine at 37°C. Cell samples were collected, centrifuged at 2,000*g* and prepared for fluorescence microscopy as described in [57]. Fluorophores were visualized using band-pass CFP (31044) and RFP (41002c) filter sets from Chroma. Fluorescence images were acquired and processed using Volocity software (PerkinElmer). Statistical probabilities were calculated using Fisher’s exact test (two-tailed). See S1 Table for morphology of cells included in the study.

## Supporting Information

S1 Fig Fob1-dependent ssDNA-containing structures are formed in *sgs1Δtop2* ^*ts*^ cells in late S/G2. wt, *sgs1Δ*, *top2* ^*ts*^ and *sgs1Δtop2* ^*ts*^ cells (A) or wt, *fob1Δ*, *sgs1Δtop2* ^*ts*^, and *sgs1Δtop2* ^*ts*^ *fob1Δ* cells (B) having the endogenous Rfa1 protein (large subunit of RPA) tagged with CFP and Nop1-RFP expressed ectopically, were treated as in Fig 1A. At the indicated time points after release from α-factor, samples were withdrawn and fluorescent microscopy images were taken and analyzed. (A) Upper panel, Percentage of cells with Rfa1 foci. Middle panel, Number of Rfa1 foci per cell. Lower panel, Cellular localization of Rfa1 foci at the 80 minute time point. *in*, *peri*, and *out* indicate position of foci inside nucleolus, at the nucleolar periphery, and outside nucleolus, respectively. (B) Upper panel, Percentage of cells with Rfa1 foci. Lower panel, Number of Rfa1 foci per cell. Error bars represent 95% confidence intervals (n = 100–200). Two biological replicates.
(EPS)

S2 Fig *sgs1Δtop2* ^*ts*^ cells do not show X-spike formation at a replication termination site outside the rDNA locus. Genomic DNA was isolated from wt and *sgs1Δtop2* ^*ts*^ cells at the indicated time points after release from α-factor, digested with *Hin*dIII and subjected to 2D gel analysis and Southern blotting using a probe recognizing *TER102* on chr. I [8]. FACS profiles are shown to the right.
(EPS)

S3 Fig Checkpoint activating DNA structures are resistant to Top2 and Top3 in contrast to structures responsible for X-spike formation. (A) Outline of the experimental setup. *sgs1Δtop2* ^*ts*^ (B and C) or *top2* ^*ts*^ *top3* ^*ts*^ cells (D and E) were synchronized in G1 by α-factor at 25°C, released from α-factor into nocodazole (noc) at 34°C (37°C for *top2* ^*ts*^ *top3* ^*ts*^) and then kept at this temperature or transferred to 25°C either 25’ or 60’ after release from α-factor to reactivate Top2 (B and C) or Top2 and Top3 (D and E). Samples were withdrawn at the indicated time points and processed for ISA analysis of Rad53 (B and D) or 2D gel electrophoresis and Southern blotting with probe P1 (C and E). In (C) and (E) quantifications of Xs to Ys at the indicated time points are shown, where the Xs/Ys obtained at time point 100 in cells with no reactivation is set to 100%. Error bars represent STDEV from two independent experiments. FACS profiles are shown throughout.
(EPS)

S4 Fig Checkpoint activation does not occur due to an inability to solve HR structures. ISA analysis of Rad53 was performed as in Fig 1 with *rad52* ***Δ***, *sgs1Δtop2* ^*ts*^, and *top2* ^*ts*^ *rad52* ***Δ*** cells at the indicated time points after release of cells from α-factor. The positive controls represent ISA analysis performed on extract from MMS-treated wt cells. Mcm2 was used as a loading control. FACS profiles of samples taken throughout the experiments are shown below each strain.
(EPS)

S5 Fig HR independent X-spikes are formed in the *Bgl*IIA fragment. Genomic DNA was isolated from *sgs1Δtop2* ^*ts*^ *rad52Δ* cells at the indicated time points after release from α-factor and subjected to 2D gel analysis and Southern blotting after digestion with *Bgl*II. P2, recognizing the *Bgl*IIA fragment was used as probe. FACS profiles are shown to the right.
(EPS)

S6 Fig Chr. XII migration is restored in *sgs1Δtop2* ^*ts*^ *pif1Δ* cells. (A) The experimental setup was as shown in Fig 1A, except that cells were released at 34°C into nocodazole to block further cell cycling. Chromosomes were prepared from *sgs1Δtop2* ^*ts*^ *pif1Δ* cells at the indicated time points after release from α-factor and visualized after pulsed-field gel electrophoresis by EtBr staining (upper panel) and by Southern blotting with a probe (P1) specific for chr. XII (lower panel). *M*, Chromosomal marker with indication of individual chromosomes to the left. (B) Genomic DNA was isolated from *pif1Δ* cells at the indicated time points after release from α-factor and subjected to 2D gel analysis and Southern blotting after digestion with *Bgl*II. P1, recognizing the *Bgl*IIB fragment was used as probe. FACS profiles are shown to the right.
(EPS)

S1 Table Morphology of cells included in the fluorescence microscopy study. Cell morphology indicates cell cycle phase. Only cells that were synchronized in G1 with visible shmoo formation at 0’ and progressed into budded cells after release into the S phase were counted. The increase in number of cells with foci coincides with the appearance of buds for all strains, indicating that foci are formed due to active replication.
(DOCX)

S2 Table Strains used in this study. All strains are derivatives of W303-1a.
(DOCX)
