# Supplementary figures and images for: Top2 and Sgs1-Top3 Act Redundantly to Ensure rDNA Replication Termination
Source: PLoS Genet. 2015 Dec 2;11(12):e1005697. doi: 10.1371/journal.pgen.1005697 (PMC4668019; doi:10.1371/journal.pgen.1005697)

**
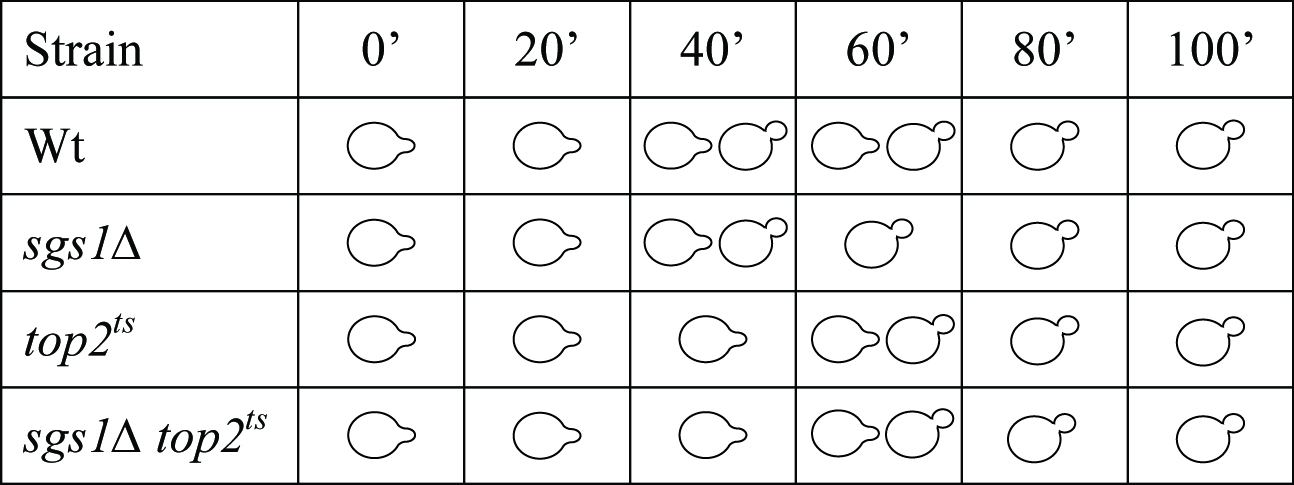
**

Supplement: S1 Table — Cell morphology indicates cell cycle phase. Only cells that were synchronized in G1 with visible shmoo formation at 0’ and progressed into budded cells after release into the S phase were counted. The increase in number of cells with foci coincides with the appearance of buds for all strains, indicating that foci are formed due to active replication. (DOCX) [file pgen.1005697.s007.docx]
