# Supplementary material for: Top2 and Sgs1-Top3 Act Redundantly to Ensure rDNA Replication Termination
Source: PLoS Genet. 2015 Dec 2;11(12):e1005697. doi: 10.1371/journal.pgen.1005697 (PMC4668019; doi:10.1371/journal.pgen.1005697)
Supplement: S2 Table — All strains are derivatives of W303-1a. (DOCX) [file pgen.1005697.s008.docx]

| Strain (Ay-) | Genotype | Source |
| --- | --- | --- |
| 107 | Ay-120 with *top2-1^ts^, sgs1*::*TRP1* | This study |
| 120 | *MATa, ade2-1, trp1-1, his3-11, -15, ura3-1, leu2-3, -112, can1-100* | R. Rothstein |
| 124 | Ay-120 with *sgs1*::*TRP1* | This study |
| 127 | Ay-120 with *top2-1^ts^* | R. Sternglanz |
| 176 | Ay-120 with *rad52::LEU2 529* | This study |
| 185 | Ay-120 with *rad52::LEU2 529, sgs1::TRP1* | This study |
| 217 | Ay-120 with *mec1::HIS3, sml1::URA3* | This study |
| 218 | Ay-120 with *mec1::HIS3, sml1::URA3, sgs1::TRP1* | This study |
| 219 | Ay-120 with *mec1::HIS3, sml1::URA3, top2-1^ts^* | This study |
| 220 | Ay-120 with *mec1::HIS3, sml1::URA3, sgs1::TRP1, top2-1^ts^* | This study |
| 227 | Ay-120 with *fob1::URA3* | This study |
| 228 | Ay-120 with *fob1::URA3, sgs1::TRP1* | This study |
| 229 | Ay-120 with *fob1::URA3, sgs1::TRP1, top2-1^ts^* | This study |
| 273 | Ay-120 with *rad52::LEU2 529, sgs1::TRP1, top2-1^ts^* | This study |
| 275 | Ay-120 with *rad52::LEU2 529, top2-1^ts^* | This study |
| 319 | Ay-120 with RAD52-YFP, RFA1-8ala-CFP, transformed with pWJ1321 | This study |
| 322 | Ay-120 with *sgs1::TRP1,* RAD52-YFP, RFA1-8ala-CFP, transformed with pWJ1321 | This study |
| 325 | Ay-120 with *top2-1^ts^,* RAD52-YFP, RFA1-8ala-CFP, transformed with pWJ1321 | This study |
| 328 | Ay-120 with *sgs1::TRP1, top2-1^ts^,* RAD52-YFP, RFA1-8ala-CFP, transformed with pWJ1321 | This study |
| 386 | Ay-120 with *top3-E447K, S583L* | R. Rothstein |
| 388 | Ay-120 with *top2-1^ts^, top3-E447K, S583L* | This study |
| 448 | Ay-120 with RAD52-YFP, RFA1-8ala-CFP, *fob1::URA3* | This study |
| 449 | Ay-120 with RAD52-YFP, RFA1-8ala-CFP, *fob1::URA3, sgs1::TRP1, top2-1ts,* | This study |
| 461 | Ay-120 with *rad51::URA3, sgs1::TRP1, top2-1^ts^* | This study |
| 468 | Ay-120 with *pif1::HIS3, sgs1::TRP1, top2-1^ts^* | This study |
